# Chest CT features of patients under investigation for Covid-19 pneumonia in a Ghanaian tertiary hospital: a descriptive study

**DOI:** 10.4314/gmj.v54i4.8

**Published:** 2020-12

**Authors:** Klenam Dzefi-Tettey, Patience S Saaka, Isaac Acquah, Emmanuel K M Edzie, Philip N Gorleku, Patrick Adjei, Jonathan K Semetey, Edward K D Ayem, Arwen J Insaidoo, Ali Samba

**Affiliations:** 1 Department of Radiology, Korle Bu Teaching Hospital, Accra. Ghana; 2 Center for Outcomes Research, Houston Methodist Research Institute, Houston, TX 77030, USA; 3 Department of Medical Imaging. School of Medical Sciences, College of Health and Allied Sciences, University of Cape Coast, Cape Coast. Ghana; 4 Department of Medicine and Therapeutics, University of Ghana Medical School, College of Health Sciences, University of Ghana, Korle Bu Teaching Hospital, Accra, Ghana; 5 Korle Bu Teaching Hospital, Accra. Ghana

**Keywords:** Coronavirus disease 2019, chest, computerized tomography scan, tertiary hospital, Ghana

## Abstract

**Background:**

Coronavirus disease 2019 (COVID-19) has since December 2019 become a problem of global concern. Due to the virus' novelty and high infectivity, early diagnosis is key to curtailing spread. The knowledge and identification of chest Computerized Tomography (CT) features in Patients Under Investigation (PUI) for the disease would help in its management and containment.

**Objectives:**

To describe the chest CT findings of PUI for COVID-19 pneumonia referred to the Department of Radiology of the Korle Bu Teaching Hospital; as well as to determine the relationship between symptom onset and severity of the chest CT findings.

**Methods:**

The study was retrospective and included 63 PUI for COVID-19 referred to the Department between 11^th^ April, 2020 and 10^th^ June, 2020, for non-enhanced chest CT imaging. Clinical data were obtained from patients' records and Reverse Transcriptase-Polymerase Chain Reaction (RT-PCR) results were acquired after the CT evaluation.

**Results:**

The mean age in years was 51.1±19.9 SD. More males (52.8%) than females (47.2%) tested positive for COVID-19 and the age range for positive cases was 7 months to 86 years, with a mean of 53.2±21 SD years. Common features of COVID-19 pneumonia were bilateral posterior basal consolidations, Ground Glass Opacities (GGO) and air bronchograms. Findings were worse in patients scanned 5–9 days after onset of symptoms.

**Conclusion:**

Adequate knowledge of chest CT features of COVID-19 pneumonia, proves a valuable resource in triaging of symptomatic patients and consequent containment of the disease in the hospital setting.

**Funding:**

None declared

## Introduction

There has been a global spread of an acute viral disease, Corona Virus Disease 2019 (COVID-19), since December 2019. It has been found to be caused by the novel Severe Acute Respiratory Syndrome Corona Virus 2 (SARS-CoV-2).^1^ Like earlier pandemic-causing viruses such as SARS coronavirus (SARS-CoV) and Middle East Respiratory Syndrome Coronavirus (MERS-CoV), SARS-CoV-2 is a β-coronavirus.^2^ However, it has a higher infectivity potential than earlier encountered coronaviruses, achieving this through a higher binding affinity of its spike protein to the human angiotensin converting enzyme 2 receptor site of the respiratory epithelium of humans.^2^,^3^

The first cluster of cases were reported in Wuhan located in the Hubei province of China. It is purported to have originated from the Huanan seafood market. Person-person transmission of this severe acute respiratory syndrome was subsequently established and on March 11^th^ of 2020, it was declared a pandemic by the World Health Organization (WHO).^2^,^4^

Ghana recorded its first two cases on the 12^th^ of March 2020 approximately a month after the first case was recorded in Africa.^5^ The numbers have since increased with well over 10,200 confirmed cases and 48 deaths as of June 9^th^, 2020.^6^ Over this period, Persons Under Investigation (PUI) for COVID-19 have been referred to the Radiology Department of the Korle Bu Teaching Hospital (KBTH) for non-enhanced chest CT imaging studies to help characterise their lung diseases.

Since the outbreak of the disease, there have been various publications on the radiological presentation of patients with COVID-19, mainly carried out in China and the western world. These have shown the various patterns of lung involvement on chest CT.^1^,^7^–^9^ Thus, have served as benchmarks for the characterisation of radiological (particularly chest CT) findings for COVID-19.

With the ever-evolving nature of the virus and disease, it is imperative that chest CT imaging findings of cases seen in our part of the world are documented and possibly compared with findings from China and the western world to fill in the knowledge gap and add to the growing body of knowledge.

Our aim was to describe the non-enhanced chest CT imaging findings and to determine the relationship between time of onset of symptoms and severity of non-enhanced chest CT imaging findings of PUI for COVID-19 pneumonia seen at the department of radiology of the KBTH.

## Methods

### Study design, setting and participants

This was a retrospective descriptive study carried out at the Radiology Department of the Korle Bu Teaching Hospital (KBTH), a tertiary hospital located in Accra, the capital of Ghana. Established in 1923, this 2000-bed capacity hospital is currently the third largest hospital in Africa and the leading referral center for Ghana.^10^ The Radiology Sub-Budget Management Committee (Sub-BMC) 2019 annual report revealed that the department performs over 1,080 chest CT scans per year. Approval for this study was given by the KBTH Institutional Review Board [KBTH-STC-00108/2020].

We reviewed 63 PUI for COVID-19 pneumonia (also known as suspected cases of COVID-19) referred to the department of radiology between the 11^th^ of April 2020 and the 10^th^ of June 2020 for non-enhanced chest CT scans. A suspected case of COVID-19 was defined as an individual who met at least three of the following criteria: fever, anosmia, rhinorrhoea, cough, dyspnoea, myalgia, abnormal chest findings on physical examination, pneumonia not responding to empirical antibiotic agents, and a history of travel from other countries with recorded cases of COVID-19 or an epidemiologic link with a COVID-19 patient. All cases underwent RT-PCR testing of throat swab specimen to confirm or exclude COVID-19, results of which were obtained post-CT evaluation. Clinical data such as symptoms, time of onset and laboratory findings were obtained from patients' clinical notes with all information de-identified to preserve patient anonymity and confidentiality. The patients were selected consecutively with no exclusions made.

### CT Image acquisition

All the non-enhanced chest CT images were acquired using a 32-slice Canon AquillionStart (model TSX-037A, 2019) Multi-Detector CT scanner (Otawara, Tochigi, Japan). Images were taken in a single breath-hold at endinspiration (to help reduce motion artefacts), with patient in the supine position. Scanning was from the level of the upper thoracic inlet to the inferior-most level of the costophrenic angle. The parameters used were: tube voltage 120 kV; tube current-exposure time 300mAs; matrix 512 × 512, slice thickness and interval 1mm and 0.625mm respectively. Images were subsequently reconstructed at the workstation and transferred to the Picture Archiving and Communication System (PACS) (IBM Watson Health Global Headquarters, Cambridge, MA, USA).

### Image interpretation

The images were reviewed by three radiologists with over 10 years of experience in interpreting chest CT images. Where there were disagreements between reports, unanimity was reached by discussion. Reporting was done following the Radiological Society of North America (RSNA) Expert Consensus Statement on Reporting Chest CT Findings Related to COVID-19.^11^ Findings were described under three main brackets namely; pleural, lung and bronchial changes and lesions were defined based largely on the Fleischner Society Glossary of terms for thoracic imaging.^7^,^12^

Lung features were described as the presence or absence of GGO which appears as a hazy increased opacity of lung, with preservation of bronchial and vascular margins), crazy paving pattern (appear as thickened interlobular septa and intra-lobular lines superimposed on a background of ground glass opacity resembling irregularly shaped paving stones), consolidation (a homogeneous increase in pulmonary parenchymal attenuation that obscures the margins of vessels and airway walls), reversed halo sign (a focal round area of ground glass opacity surrounded by a more or less complete ring of consolidation), cavitation (a gas-filled space or low-attenuation area within pulmonary consolidation, a mass or a nodule), nodule (rounded or irregular opacity, well or poorly defined, measuring up to 3 cm in diameter), micronodule (a discrete small, round, focal opacity with diameter no greater than 7 mm), and septal thickening (thin linear hyperdensities at right angles to and in contact with the lateral pleural surfaces near the lung bases).^13^–^15^ Opacity patterns were classified as predominantly ground glass, predominantly consolidation or predominantly nodular if the percentage of the particular pattern was greater than 50%.^13^,^14^ Based on distribution, these opacities were further classified as peripheral, central or mixed (peripheral and central), anterior or posterior and the lobes involved noted. Pleural changes were defined by the presence or absence of pleural thickening or pleural effusions. Bronchial changes were defined by the presence or otherwise of air bronchogram (a pattern of low-attenuation bronchi on a background of high attenuation airless lung), (identifying features of which include bronchial dilatation with respect to the accompanying pulmonary artery/signet ring sign, lack of tapering of bronchi and identification of bronchi within 1 cm of the pleural surface).^12^, ^15^ Pneumothorax was also looked out for and documented.

The duration of onset of symptoms was categorised into 4 stages: stage 1 (0–4 days), stage 2 (5–9 days), stage 3 (10–14 days) and stage 4 (15–21 days). The extent of lobar involvement with respect to the duration of onset of symptoms was evaluated. Based on the RSNA Expert Consensus Statement on Reporting Chest CT Findings Related to COVID-19, each non-enhanced chest CT scan report was classified as: Typical appearance for COVID-19 pneumonia, Indeterminate appearance, Atypical appearance or Negative for pneumonia.^11^ Under this consensus statement, a PUI for COVID-19 has Typical features when there are multifocal rounded ground glass opacities with consolidation in a peripheral distribution with scattered areas of intralobular lines (“crazy paving”) and a “reversed halo” sign but without a pleural effusion or pneumothorax.

The features are Indeterminate when there are multifocal non-rounded ground glass opacities and consolidations with diffuse, perihilar or unilateral distribution but without peripheral distribution. The features are assigned as Atypical for COVID-19 pneumonia if they show multifocal tree-in-bud opacities, isolated lobar or segmental consolidation, discrete small nodules (tree-in-bud), lung cavitation, or smooth interlobular septal thickening with pleural effusion (oedema). Lastly, a patient is Negative for COVID-19 pneumonia when there are no obvious lung parenchymal lesions.

### Statistical analyses

Data were analysed using STATA 16.1 (StataCorp, College Station, Texas, USA). Quantitative data were represented as mean ± standard deviation (SD) while frequencies of CT features were represented as percentages. Tables, graphs and charts were constructed with Microsoft Excel 2010 (Microsoft Corp., Redmond, WA, USA). For all statistical analyses, p≤0.05 was considered statistically significant. The Shapiro-Wilk test was used to test for normality and continuous variables were compared using the Mann-Whitney U test. Differences in categorical variables were compared using the Fisher's exact test.

## Results

Table 1 summarises the demographic and clinical characteristics of the patients studied.

**Table 1:** General characteristics and clinical features of PUI for COVID-19 pneumonia

	All	Positive RT-PCR	Negative RT-PCR	p-values
Total number	63	36	24	
Age (mean), years	51.1 ± 19.9	53.2 ± 21.0	47.3 ± 18.6	
Age group				0.64
**<18 years**	4 (6.7)	3 (8.3)	1 (4.2)	
**≥ 18 years**	56 (93.3)	33 (91.7)	23 (95.8)	
Sex			1.00	
**Male**	34 (54.0)	19 (52.8)	13 (54.2)	
**Female**	29 (46.0)	17 (47.2)	11 (45.8)	
Symptoms				
**Fever**	33 (70.2)	17 (65.4)	14 (77.8)	0.51
**Cough**	33 (67.4)	18 (60)	13 (81.3)	0.20
**Anosmia**	21 (41.2)	14 (46.7)	7 (36.8)	0.56
**Dyspnoea**	38 (65.5)	22 (66.7)	16 (68.2)	1.00
**Myalgia**	22 (41.5)	15 (50)	6 (28.6)	0.16
**Headache**	12 (25.5)	9 (33.3)	2 (11.1)	0.16
**Abdominal symptoms**	10 (24.4)	7 (26.9)	3 (21.4)	1.00
Symptom onset (mean), days	7	7.8	6.4	0.06
Travel history			-	
**Recent travel**	0 (0.0))	0 (0.0)	0 (0.0)	
Exposure history			0.51	
**Infected contact**	2 (3.2)	2 (5.6)	0 (0.0)	
**Unknown**	61 (96.8)	34 (94.4)	24 (100)	
Laboratory results				
**Leucocytes**				0.51
**Decreased**	4 (11.8)	3 (13.6)	1 (8.3)	
**Normal**	25 (73.5)	17 (77.3)	8 (66.7)	
**Increased**	5 (14.7)	2 (9.1)	3 (25.0)	
**Lymphocytes**				0.57
**Decreased**	9 (27.3)	7 (31.8)	2 (18.2)	
**Normal**	20 (60.6)	13 (59.1)	7 (63.6)	
**Increased**	4 (12.1)	2 (9.1)	2 (18.2)	
**CRP**				0.64
**Normal**	7 (26.9)	4 (22.2)	3 (37.5)	
**Increased**	19 (76.1)	14 (77.8)	5 (62.5)	
**ESR**				1.00
**Normal**	2 (9.5)	1 (7.1)	1 (14.3)	
**Increased**	19 (90.5)	13 (92.9)	6 (85.7)	

Note: The counting data are presented as count (percentage). RT-PCR-Reverse Transcriptase Polymerase Chain Reaction. ESR- Erythrocyte Sedimentation Rate. CRP- C-Reactive Protein.

A total of 63 patients were evaluated in this study. Thirty-four (54%) were males and 29 (46%) females. Of the 63, 36 tested positive for SARS-CoV-2 and 24 tested negative. The mean age in years of the study population was 51.1±19.9. The mean age of patients who tested positive was 53.2±21 SD years with an age range of 7 months to 86 years with more males (52.8%) than females (47.2%). Symptoms of dyspnoea, cough, fever, anosmia and myalgia dominated in both positive and negative individuals. Two (5.6%) patients who tested positive admitted to being in contact with an infected person while 34 (94.4%) did not know their exposure history. Leucocyte and lymphocyte counts were predominantly normal across both groups. The positive group had 13.6% of evaluated cases having leucopoenia and 9.1% of them had leukocytosis. Of the positive cases whose C-reactive protein (CRP) levels were evaluated, 14 (77.8%) had increased levels with 4 (22.2%) being normal. Similarly, 13 (92.9%) of the 14 positive cases evaluated for erythrocyte sedimentation rate (ESR) had increased values.

None of the patients in the negative group had the reversed halo or crazy paving signs (Table 2).

**Table 2:** Chest CT findings in PUI for COVID-19 pneumonia

	All	Positive RT-PCR	Negative RT-PCR	p-values
Lung Features				
**GGO**	28 (45.9)	19 (52.8)	8 (36.4)	0.28
**Consolidation**	45 (73.8)	26 (72.2)	17 (77.4)	0.76
**Crazy paving**	6 (9.8)	6 (16.7)	0 (0.0)	0.07
**Micronodules**	9 (14.8)	6 (16.7)	2 (9.1)	0.70
**Reversed halo**	1 (1,6)	1 (2.8)	0 (0.0)	1.00
**Cavitation**	7 (11.5)	2 (5.6)	3 (13.6)	0.36
**Septal thickening**	8 (13.1)	7 (19.4)	1 (4.6)	0.14
**No lesion**	10 (15.9)	6 (16.7)	4 (16.7)	0.17
Pleural features				
**Thickening**	18 (29.5)	11 (30.6)	5 (22.7)	0.56
**Effusion**	16 (26.2)	10 (27.8)	5 (22.7)	0.76
Bronchial features				
**Air bronchogram**	36 (59.0)	22 (61.1)	12 (54.6)	0.78
**Bronchiectasis**	6 (9.8)	3 (8.3)	3(13.6)	0.66
Lesion distribution				
**Peripheral**	33 (54.1)	23 (61.9)	8 (36.4)	0.06
**Central**	4 (6.6)	3 (8.3)	1 (4.6)	1.00
**Mixed**	20 (32,8)	11 (29.9)	8 (36.4)	0.78
**Anterior**	13 (21.3)	6 (16.7)	5 (22.7)	0.73
**Posterior**	45 (73.8)	29 (80.6)	13 (59.1)	0.13
Lesion predominance				0.16
**GGO**	13 (21.3)	10 (27.8)	2 (9.1)	
**Consolidation**	32 (52.5)	16 (44.4)	15 (68.2)	
**Nodular**	4 (6.6)	3 (8.3)	0 (0.0)	
Pneumothorax	0 (0.0)	0 (0.0)	0 (0.0)	-
Lymphadenopathy				
**Mediastinal**	0 (0.0)	0 (0.0)	0 (0.0)	-
**Hilar**	0 (0.0)	0 (0.0)	0 (0.0)	-

Note: The counting data are presented as count (percentage).

Air bronchograms were the most common bronchial features, found in 22 (61%) of positive cases and 12 (54.6%) of negative cases. Ten patients in total had no lung parenchyma lesions of which 6 were in the positive group and four in the negative group. Lesions were mostly distributed peripherally and posteriorly and were predominantly consolidations. Pneumothorax was not noted in any of the two groups. There were no significant differences seen in the chest CT findings between adults and children.

Table 3 shows the comorbid conditions in patients who tested RT-PCR positive for COVID-19. Of the 36 patients in this group, 72.2% had comorbid conditions with hypertension (61.1%) ranking high as the most common comorbid condition. One out of the 4 individuals in the “< 18 years” age group had a comorbid condition.

**Table 3:** Comorbid conditions in RT-PCR (COVID-19) patients

Comorbidities	RT-PCR Positive
**Hypertension**	22 (61.1)
**Diabetes**	15 (41.7)
**Chronic pulmonary diseases**	4 (11.1)
**Congestive cardiac failure**	4 (11.1)
**Cerebrovascular disease**	0 (0.0)
**Chronic renal failure**	5 (13.9)
**Retroviral infection**	2 (5.6)
**Malignancy**	2 (5.6)
**Number of Comorbidities per individual**	
**None**	10 (27.8)
**1**	9 (25.0)
**2**	12 (33.3)
**> 2**	5 (13.9)

**Figure 1:**
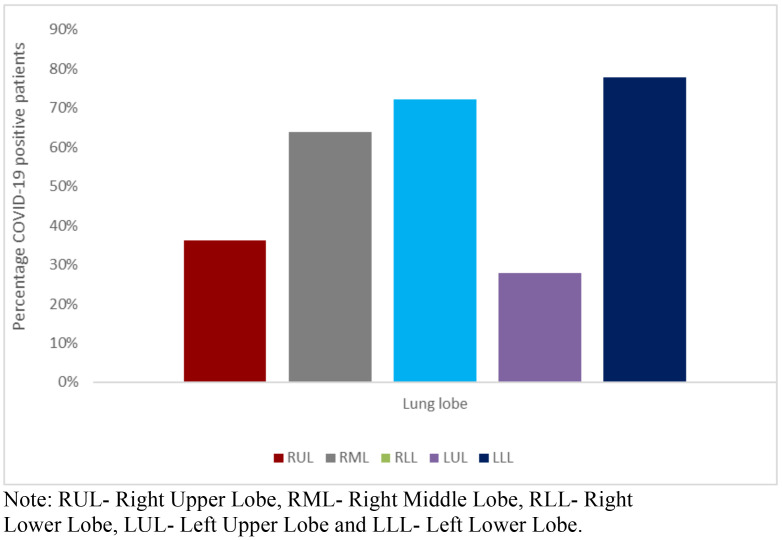
Frequency of individual lobes with disease in positive RT-PCR (COVID-19) patients. Note: RUL- Right Upper Lobe, RML- Right Middle Lobe, RLL- Right Lower Lobe, LUL- Left Upper Lobe and LLL- Left Lower Lobe.

As represented in Figure 1 below, the left and right lower lobes had the most lobar involvement with the left (77.8%) slightly more than the right (72.2%).

The left upper lobe was the least affected (27.8%). Cumulatively, the right lung was affected more compared to the left lung.

**Figure 2:**
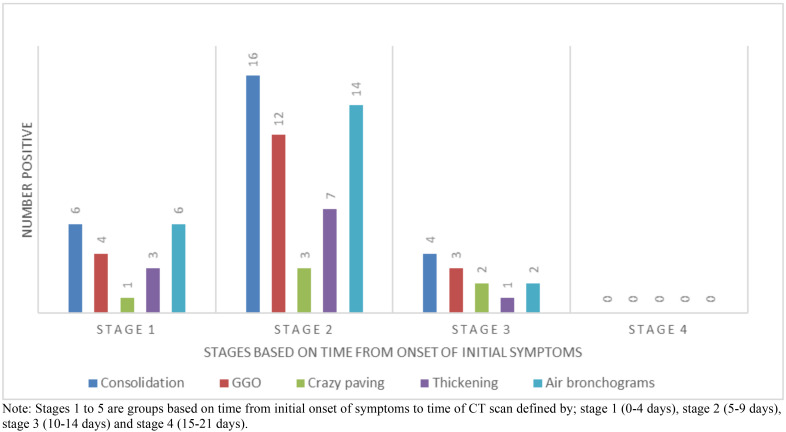
Predominant patterns of abnormality on CT imaging with time of onset in patients with positive RT-PCR (COVID-19 pneumonia). Note: Stages 1 to 5 are groups based on time from initial onset of symptoms to time of CT scan defined by; stage 1 (0–4 days), stage 2 (5–9 days), stage 3 (10–14 days) and stage 4 (15–21 days).

On plotting the predominant patterns with the time of onset of symptoms (Figure 2), we found that stage 2 (5–9 days after symptom onset) marked the highest point of lung disease in patients with COVID-19 pneumonia. None of the patients scanned in stage 4 (>14 days after onset of symptoms) had consolidation, GGOs, crazy paving, pleural thickening or air bronchograms.

**Figure 3:**
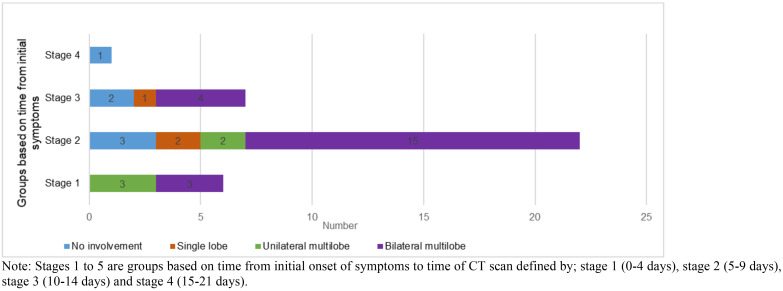
Extent of lobar involvement with time of onset of symptoms on chest CT imaging in patients with positive RT-PCR (COVID-19 pneumonia). Note: Stages 1 to 5 are groups based on time from initial onset of symptoms to time of CT scan defined by; stage 1 (0–4 days), stage 2 (5–9 days), stage 3 (10–14 days) and stage 4 (15–21 days).

Bilateral multilobar lung disease was the commonest finding as depicted in Figure 3. Patients scanned in stage 1 had 3 people having unilateral multilobar disease and 3 with bilateral multilobar lung involvement. In stage 2, 15 had bilateral multilobar disease, 2 had unilateral multilobar disease, 2 had involvement of a single lobe and 3 had no pneumonia at all. In stage 3, 4 had bilateral multilobar disease, none had unilateral multilobar disease, 1 had involvement of a single lobe and 2 had no involvement of the lobes. Patients scanned in stage 4 had no lobar involvement.

**Figure 4:**
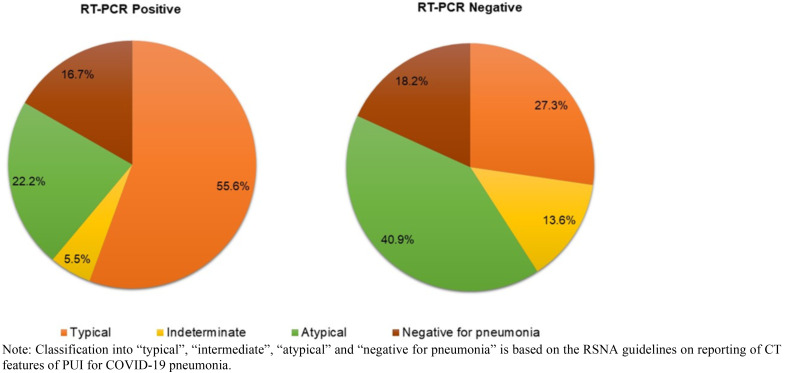
Classification of chest CT features in PUI for COVID-19 pneumonia using RSNA consensus statement. Note: Classification into “typical”, “intermediate”, “atypical” and “negative for pneumonia” is based on the RSNA guidelines on reporting of CT features of PUI for COVID-19 pneumonia.

Based on the RSNA consensus statement for standardised reporting of CT features of PUI for COVID-19 pneumonia, the RT-PCR positive group, had 55.6% of Typical features for COVID-19 pneumonia (Figure 4), 22.2% had Atypical features, findings were Indeterminate in 5.5% and 16.7% were Negative for pneumonia. In the RT-PCR negative group, the majority (40.9%) had findings Atypical for COVID-19 pneumonia. 27.3% had patterns Typical for COVID-19 pneumonia with 13.6% demonstrating Indeterminate features and the rest (18.2%) were Negative for pneumonia.

Figures 5,6,7,8,9,10,11 and 12 shows some of the Chest CT features described in our patients.

**Figure 5:**
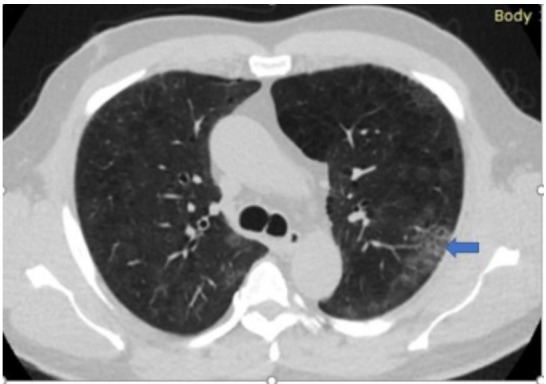
An axial non-enhanced chest CT scan of a 62-year-old male with fever and cough of 3-days duration. Lung window image shows “crazy paving pattern” (interlobular & intralobular septal thickening) in the periphery of the left lower lobe (arrow) and subtle patchy bilateral ground-glass opacities. Was found to be RT-PCR positive for COVID-19.

**Figure 6:**
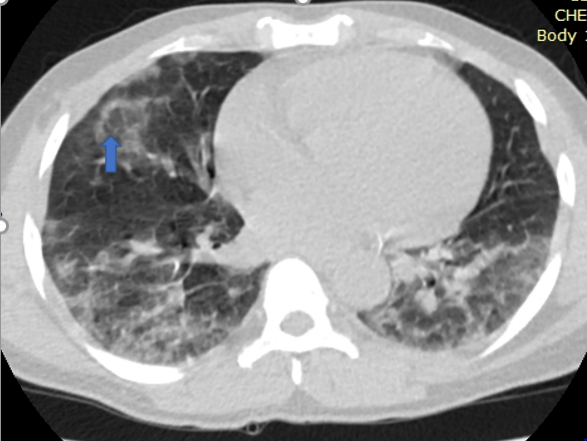
An axial non-enhanced chest CT scan of a 52-year-old man with breathlessness and cough of 14-days duration. Lung window image shows a reversed halo sign (arrow) with bilateral posterior basal consolidation. RT-PCR test confirmed COVID-19.

**Figure 7:**
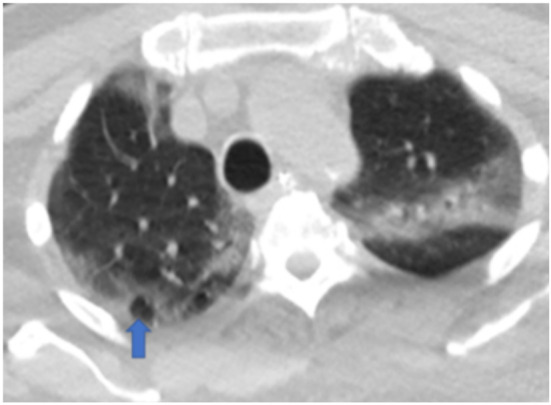
An axial non-enhanced chest CT scan, lung window of a 76-year-old man with a 5-day history of fever shows right peripheral ground glass infiltrates and cavitation (Arrow). Consolidation on the left. RT-PCR test was positive.

**Figure 8:**
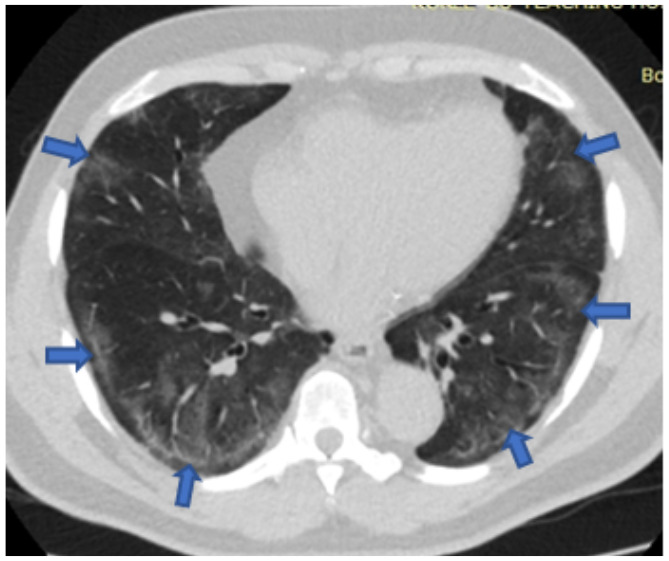
An axial non-contrast enhanced chest CT scan of a 62-year-old man with fever and breathlessness for 5 days. Lung window image shows diffuse bilateral ground glass opacities (Arrows) in a predominantly peripheral distribution. Typical features. RT-PCR confirmed COVID-19.

**Figure 9:**
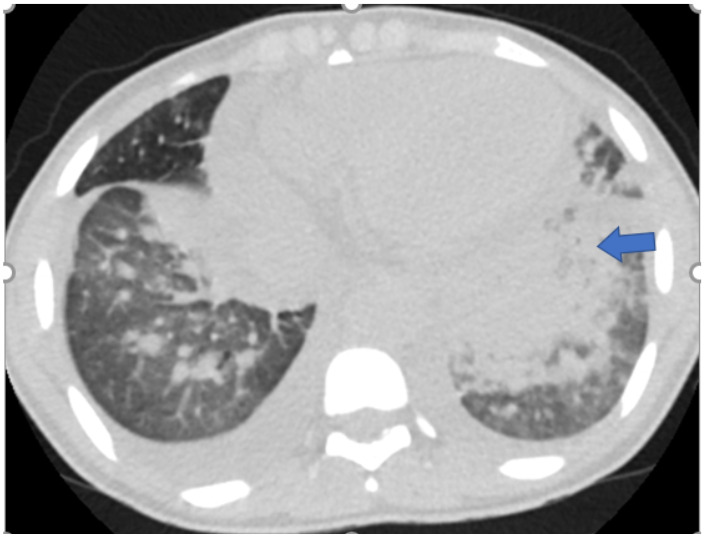
An axial non-contrast enhanced chest CT scan; lung window of a 66-year-old woman with fever and breathlessness for 9-days shows diffuse, bilateral, posterior basal, ground glass infiltrates, dense consolidation (Arrow) and air bronchograms with minimal right pleural effusion. Typical features. RT-PCR confirmed COVID-19.

**Figure 10:**
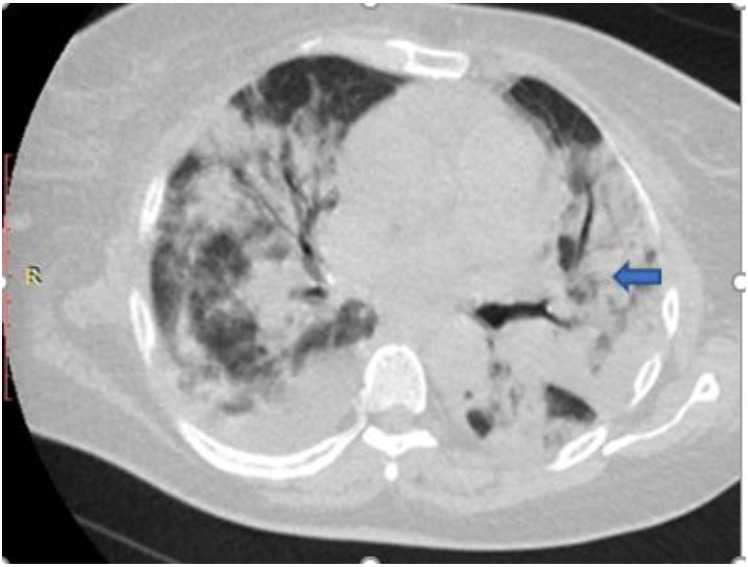
An axial non-contrast enhanced chest CT scan; lung window of a 9-year-old girl with a 10-day history of fever and cough shows dense consolidation (Arrow) in the left lower lung zone, which is not peripherally distributed but instead, more central. Tiny multifocal consolidations and ground glass infiltrates are seen on the right along with a minimal right pleural effusion. Features were categorised as ‘Indeterminate’. RT-PCR, however, confirmed COVID-19.

**Figure 11:**
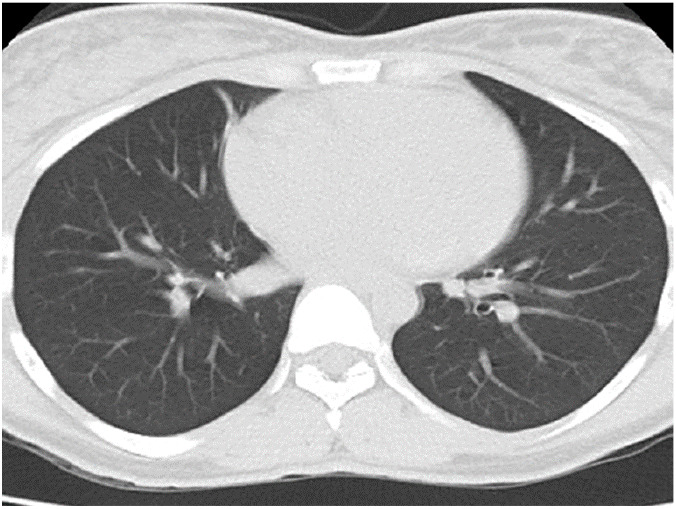
An axial non-contrast enhanced chest CT scan of a 36-year-old female with severe respiratory distress, fever, cough and anosmia of 9 days duration. Lung window image shows no parenchymal abnormality. Patient tested positive for COVID-19 but had (normal) Negative chest CT features throughout the course of the disease.

**Figure 12:**
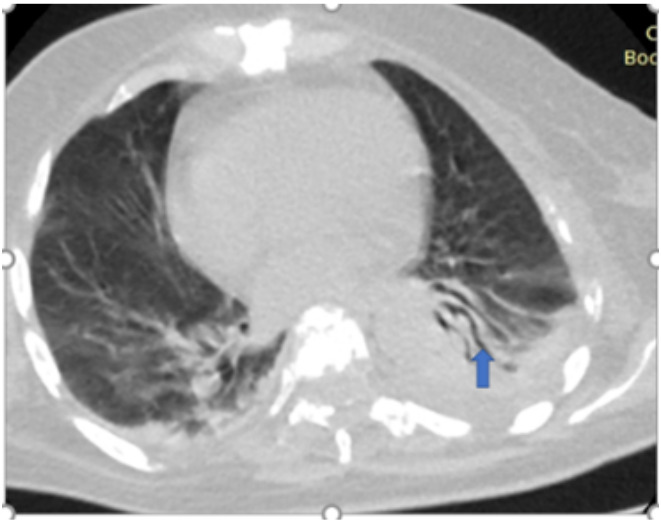
57- year -old female, breast cancer patient had fever, chest pain and cough for 9 days, Axial non-contrast enhanced CT Scan of the chest, lung window showed left segmental consolidation with air bronchograms (Arrow), minimal pleural effusion and minimal consolidation in the right lower lung zone. No peripheral distribution. Categorised as Atypical. Also noted is lytic destruction (metastatic) of the thoracic vertebral body. RT-PCR was positive, but the imaging features are not Typical and could represent pneumonia related to COVID-19 or a secondary infectious process.

## Discussion

In response to the growing numbers of COVID-19 cases in our hospital, country and across the world, we performed this study to describe the non-contrast enhanced chest CT imaging features in PUI for COVID-19 pneumonia within our context. The knowledge of these findings will aid in speedy identification of the Typical and Atypical features of COVID-19 pneumonia and thus contribute immensely to patient management and control of spread. According to Rubin *et al.*, in addition to being sensitive in identifying early parenchymal lung lesions, chest CT is also very beneficial in detecting disease progression as well as several other differential diagnoses.^16^ Being one of the most readily available imaging modalities used in radiological practice in Ghana, its role in imaging of PUI for COVID-19 cannot be downplayed.^17^

Our study showed that, out of the 63 persons investigated, 36 had a positive RT-PCR test result for SARSCov-2, 24 were negative and the results for three individuals were not available to us. The mean age of patients who tested positive was 53.2±21 years within an age range of 7 months to 86 years, suggesting that the virus has the ability to infect and cause disease in people across the age spectrum with increased rates in the elderly.^18^,^19^ More men (52.8%) tested positive for the virus than women (47.2%). Several studies have described a similar prevalence of the disease among men and women with men shown to have an increased risk for worse outcomes than women.^20^,^21^

Consistent with several other studies, the most frequently occurring symptoms were dyspnoea, cough and fever in patients with positive RT-PCR results, suggesting a predilection of the virus for the lower airway.^19^, ^22^–^25^ Myalgia and anosmia were also seen to occur commonly in these patients. Other non-specific symptoms such as headache and abdominal symptoms occurred less frequently. While these symptoms may overlap greatly with several other disease conditions, it is expedient that they are probed with a high level of suspicion in suspected cases of COVID-19 in order to curtail spread.

In terms of exposure, none of the cases recorded had a positive travel history suggesting a high rate of community spread. Of the 2 cases who indicated contact with an infected person, both tested positive for the virus supporting studies which have demonstrated the high infectivity of the SARS-CoV-2.^26^ That, 94.4% of confirmed cases did not know their exposure history suggests ubiquity of the virus and community spread.

In patients with confirmed COVID-19, 77.3% of those evaluated for leucocyte count had results within normal range. 13.6% had leucopoenia with only 9.1% showing leukocytosis. Similarly, only 9.1% of these cases had a lymphocytosis with 59.1% being normal and the remaining 31.8% having a decreased lymphocyte count. The findings in literature vary. Some report lymphocytosis in the majority of cases^19^ while others report decreased lymphocyte counts.^18^ Considering these variations, lymphocyte levels may have been influenced by the time within the disease course when patients were evaluated for these parameters. We also noted in our study that, aside a positive RT-PCR, ESR and CRP were sensitive indicators of infection.

The majority of cases in our study population had demonstrable lung parenchymal changes with consolidation and GGO as the most predominant features, which is similar to a study conducted by Sarkodie *et al.* in Ghana. However, unlike in their study, where more patients had GGO (60.9%) than consolidation (42.9%), we found consolidation (72.2%) to be more common than GGO (52.8%) in our study population.^27^ This disparity may be as a result of a smaller sample size in their study compared to ours. Crazy paving and the reversed halo signs were seen exclusively in this group albeit infrequently. Air bronchograms featured frequently with 61% of positive cases demonstrating this sign. These findings are in strong agreement with studies where these chest CT imaging features were found to be typical for COVID-19 pneumonia.^28^ Few patients had cavitations, micronodules and minimal pleural effusions, features considered atypical for pneumonia caused by SARS-CoV-2. The presence of these may signify a superimposed bacterial infection or other pathology.^28^,^29^ No pneumothorax was observed in any of the patients. The absence of pneumothorax in COVID-19 cases is in strong agreement with findings in literature.^30^ Lung lesions were mainly peripheral and adopted a posterior-basal distribution in the majority of cases, this is similar to a study of 35 confirmed cases of COVID-19 reported by Atakla *et al.* in Guinea.^31^ Both lower lobes were affected to nearly the same extent with the left lower lobe showing pathological CT features only a little more than the right lower lobe. A study by Shi *et al.*^19^ which evaluated 81 confirmed cases of COVID-19 revealed that, the right lower lobe was more affected than the left and was explained by the anatomical differences that exist between them.

We are unable to draw any strong inferences on right lower lobe versus left lower lobe predilection as the number of confirmed COVID-19 cases in our study was less than half that of Shi *et al.* We however found that the right lung was cumulatively more affected than the left. In evaluating the presence of the predominant CT abnormalities with time (between onset of symptoms and chest CT imaging) in confirmed cases of COVID-19, we realised that abnormalities were more manifested in the group with onset of symptoms between 5–9 days and least in those between 10–14 days. Further, we found that bilateral multi-lobar disease was more common among those we evaluated between 5–9 days of symptom onset. Multilobar involvement after being at its peak in the 5–9 days symptom onset group appeared to decline in patients with onset of symptoms 10 days and beyond. In a study by Pan *et al.*^32^ to investigate lung changes of COVID-19 pneumonia with time, it was highlighted that the degree of lung involvement increased with time reaching a peak between 9–13 days after onset of symptoms and steadily declined thereafter. The decline being largely as a result of resolution of lesions. This generally explains our findings. However, the differences in the time period within which the maximum CT abnormalities are found may suggest several factors among which is a probably faster progression of lung disease in our study population compared to the study earlier cited, an area which may need further investigation.

As part of our study, we classified each patient's chest CT imaging findings as Typical, Atypical, Indeterminate and Negative for COVID-19 pneumonia using the RSNA Expert Consensus Statement on Reporting Chest CT Findings Related to COVID-19.^11^,^29^ We then matched these with the RT-PCR results once they were made available. We found that, for the RT-PCR positive group, most patients displayed typical features of COVID-19 pneumonia on chest CT. In the RT-PCR negative group, atypical findings predominated. We also found that 27.3% of patients in the negative group showed typical features for COVID-19 pneumonia. Throat samples for RT-PCR testing have been found to have a positive rate of 30 – 60%.^26^,^33^ Sensitivity of the test is also low in early disease.^25^,^29^,^34^ Based on these reasons, it is possible that patients who tested negative may, in actual fact, have had the SARS-Cov-2 infection. It has therefore been advised that in cases of negative test results against a background of highly suggestive CT features, a false negative result should be considered in addition to the several other differential diagnoses under consideration.^26^,^34^,^35^ Of the patients for which follow up was possible, 3 died. All of whom had typical chest CT features for COVID-19 disease. Two of these cases had bilateral multilobar involvement whereas 1 had unilobar involvement.

The chest CT findings in these patients were not strikingly different from the features in COVID-19 pneumonia patients who survived.

The RSNA guidelines served as a valuable tool for quick reporting of non-contrast enhanced chest CT features of PUI for COVID-19 pneumonia and thus, contributing to the early containment of the disease. It is our view that Chest CT imaging, where available, proves a valuable resource in triaging of symptomatic cases in hospital settings. This is particularly true in settings where RT-PCR testing is not easily accessible or where results are slow to be made available to healthcare workers directly involved in the care of PUI for COVID-19 pneumonia. We recommend further studies to assess the evolution of CT features in patients with time as well as longitudinal studies in patients who have recovered from the disease. This will help establish long-term lung parenchymal changes post COVID-19 pneumonia.

Our study was limited by a difficulty in obtaining (and in some instances the unavailability of) patient records. Secondly, the retrospective nature of our study made followup imaging of patients a challenge. The RT-PCR results for 3 patients could not be obtained and were therefore handled as missing values during analyses. Finally, the images acquired were non-contrast enhanced, hence lymphadenopathy and subtle vascular pathology could be missed.

## Conclusion

The common features of COVID-19 pneumonia are predominantly GGO and consolidation with air bronchograms and more exclusively, crazy paving and reversed halo signs. The features are often multilobar and bilateral with a peripheral distribution and a posterior basal predilection. The left and right lower lobes were the most affected with the right lung cumulatively more affected than the left lung. Chest CT features were worse in patients whose scans were taken 5–9 days after the onset of symptoms.
